# Extracts from an Inaugural Thesis on Yellow Fever

**Published:** 1875-04-01

**Authors:** W. H. H. Hutton

**Affiliations:** Chicago Medical College, 1875


MEDICAL EXAMINER.

A

*Semi-Monthly Journal of Medical Sciences.*

EDITED BY N. S. DAVIS, M.D., AND F. H. DAVIS, M.D.

No. VII. CHICAGO, APRIL 1, 1875. VOL. XVI.

### Original Communications.

### EXTRACTS FROM AN INAUGURAL THESIS ON YELLOW FEVER.

BY W. H. H. HUTTON, M. D., CHICAGO MEDICAL COLLEGE, 1875.

YELLOW fever is peculiar, in that it is confined to certain circumscribed geographical limits, namely: Upon land bordering upon the Atlantic Ocean, or rivers and estuaries emptying into it. While the tropical or semi-tropical regions of this division of the globe is its usual habitat, yet it has been carried and propagated as far north as Philadelphia, New York and Boston, 40°–41° N., St. Nazaire, France, 47° N., and Plymouth and Southampton, England, 50°–51° N.; as far South as Montevideo, 35° S., and is quite frequent in Rio Janeiro.

It seems, however, to occur endemically only in the West Indies, shores of the Caribbean Sea, and the eastern coast of Central America and Mexico. When it appears beyond these limits, it is not generated *de novo*, but its germs have been transported thither from those regions where it is endemic.

While within these limits, it is usually confined to cities, towns, and camps located upon the low alluvial shores of the sea, or rivers emptying into it, it sometimes penetrates far inland, as in Memphis and Shreveport, in 1873, where it manifested all its malignancy and fatality. Again, it mounts high above the sea on rocky heights, to which malaria rarely or never ascends, as at Gibraltar, 1,400 feet above the sea; Stony Point and Newcastle, Jamaica, 2,500 and 4,000 feet above the sea. It is unknown upon the western coast of North and South America, Australia, East Indies; or, in fact, any part of Asia, eastern coast of Africa, and only the Atlantic ports of Europe, especially those of Portugal and Spain.

As to its origin or cause, I believe it to depend upon the presence of animalcular germs; that these germs depend for their sustenance and propagation upon accumulations of animal matter—probably, a certain degree of heat and moisture; and, entering the human system, are capable there of propagating and multiplying, and of producing the morbid effects known as yellow fever.

A peculiarity in this disease is, that it produces its greatest effects in those of a phlegmatic temperament. By this, I mean those of a full habit, whose powers of assimilation are greater than those of elimination, and consequent accumulation of a superabundance of adipose tissue. When it attacks one of this class, his chances of recovery are small compared to those of a bilious temperament, whose powers of elimination are as great or greater than those of assimilation. Probably, upon this fact, we can account for the greater fatality also, of the disease among those who habitually indulge in alcoholic liquors, as we know the tendency of alcohol, in any of its forms, is to interfere with the process of metamorphosis and elimination, and increasing that of assimilation, as is noticeable in our habitual beer-drinkers. At any rate, whether the superabundant adipose tissue plays any important part in the sustenance and multiplication of these germs, is not for me to say, but the fact remains, that this class of individuals are especially obnoxious to the disease.

In regard to the geographical limitation of this species of animal life, we know that certain orders and species have their habitat in certain divisions of the world, and the *fiat* which has set the bounds of many known forms of animal life, beyond which they cannot live and thrive naturally, may certainly, also, have circumscribed the domain of this unknown animalcule.

That yellow fever is not referable in every instance to local malarial causes, or more nearly allied to the malarial than the continued fevers, is shown by the fact that it is not dependent upon the malaria engendered by vegetable decomposition, for its existence and propagation; that the two hardly ever co-exist; that while yellow fever seeks cities, towns, and camps where people are aggregated together, and the consequent accumulation of animal matter, malarial fevers seek the open country, where rank vegetation thrives, and stagnant waters abound; and amidst the greatest exhalations of miasm, yellow fever rarely appears. That, while yellow fever manifests itself only within a limited zone, malarial fevers are co-extensive with the world—in the jungles of the East Indies, wilds of Africa, the Pontine marshes, and the swamps of our own country, where it often takes on as fatal effects as the more dreaded yellow fever.

Again, malarial fever, even in its most severe forms, does not tend to produce that condition of the system which secures an immunity from subsequent attacks. On the other hand, yellow fever rarely or never attacks the same person the second time. In these two respects, then—dependence upon the accumulation of animal matter for its existence and propagation, and the faculty of exhausting the susceptibility of the individual in a single manifestation—it certainly appears more nearly allied to the *con-*

*tinued* than the malarial, or periodical fevers.

That it is also *contagious*, I will endeavor to show, after I have related what I personally know about it in that respect.

I come now to examine the discrepancies on these points by medical writers and teachers; some maintaining its non-contagious remittent type, others its contagious continued character; some affirming that long-continued high temperature, 82° F. for two or more months, is a necessary element; others doubting it, and so on.

Both are right—that is, they have all observed these conditions in connection with a disease they have all designated as yellow fever.

Until the researches of Louis and Jenner established the contrary, typhus and typhoid fever were supposed to be one, and the same pathological condition of the system, or modification of the same; so, with what has heretofore been known as yellow fever, and recognized under different forms of manifestation, symptoms, progress, pathological lesions and conditions, is now coming to be recognized and separated into two distinct diseases, arising from different causes, with some symptoms and pathology in common, and others of a distinctive character, namely: *specific yellow fever*, and *yellow* remittent, or hæmorrhagic malarial fever.

In both, we may have yellowness of skin, intense headache, delirium, and black vomit, but here the analogy ceases. In yellow remittent fever, the pulse is uniformly rapid, 110–130 during the pyrexia; the delirium is more common and greater; the characteristic pain in the lumbar region is slight or wanting; the urine is not albuminous; there is no tendency towards suppression; and this form of fever is observed occasionally, in all parts of the world where malaria abounds in its most concentrated form.

While ordinary ague results from a mild, diffusible form of malaria, remittent fever and congestive fever are caused by a more concentrated form of the same *materies morbi;* and yellow remittent fever, requires in addition to this for its generation, long-continued high temperature, and probably, a peculiar state of the surrounding atmosphere.

On the other hand, in specific yellow fever, after the premonitory symptoms, the pulse is remarkably slow; sometimes of a "hobbling" character; the pain in the lumbar region is excruciating, resembling that of a severe sprain or contusion; the delirium is slight, if any, in most cases (indeed, I have known of fatal cases in which the patient was perfectly conscious until the last moment); the urine is albuminous; there is always marked diminution, and a tendency toward suppression; suppression being a *fatal pathognomonic symptom*, much more so than that of black vomit.

In yellow remittent fever, after the febrile symptoms have obtained from twelve to seventy-two hours, a remission occurs, which, unless convalescence is established, as is sometimes the case, continues for several hours, when all the symptoms return in an aggravated form, during which black vomit is apt to supervene, and, if death does not follow, convalescence is slowly established, or it may assume a typhous form with slow lingering recovery, or death from exhaustion.

On the other hand, specific yellow fever runs its course in from three to eight days, and either kills the patient or ends in almost complete recovery, in an incredibly short space of time. Sometimes, within a few hours after the febrile symptoms have passed away, the patient with undiminished muscular strength, and a voracious appetite, insists upon leaving his bed and going about his business. This, however, should never be permitted, as the overwhelming influences to which the system has been subjected, leaves it in such condition, although not recognized by the patient, that any indiscretion in regard to diet and muscular effort during a few days after convalescence is established, will almost surely result in a relapse and death. Therefore, he should be kept quiet for a few days, and gradually accustom himself to ordinary diet.

During the fall and winter of 1868-9, I was in Montgomery, Alabama, and an epidemic of fever, of a severe and fatal character, occurred there, with yellowness of skin and black vomit, and death in a large percentage of cases. But the fact of its occurrence late in the season, after frost had fallen several times, together with the fact that it could not be traced to other than local causes, led the profession to pronounce it yellow remittent, or hæmorrhagic malarial fever.

I may be permitted to say here, what I ought to have said before, that in specific yellow fever, the occurrence of white frost invariably puts an end to it, at least in its epidemic form; and no one who has not witnessed it, can imagine the joy which lights up the countenances of a people afflicted with this scourge, when, on rising some cold, clear morning, they see a white mantle spread abroad on the housetops and earth. Instantly, the event long and anxiously looked, hoped, and prayed for, is dispatched to those who have fled at its approach, and they return with feelings of safety to their abandoned homes. Cold is destructive to the germs of yellow fever, and only those will suffer thereafter during that season, who have already been contaminated, or who may enter dwellings where the germs may have been developed, and may not have been subjected to such a degree of cold as to destroy them.

I come now to relate my personal experience with yellow fever, and I believe I am right when I say that the experience and observation of interested individuals, are worth more to the science of medicine than all the fine-spun theories ever elaborated from the brain of the enthusiast.

In 1867, I was stationed at Fort Gaines, on Dauphin Island, 33 miles south of Mobile. The island is from one-fourth to one mile wide, and about fifteen miles long. It is low, flat, and covered with rank vegetation and swamps. In the summer and fall months, the exhalations from these marshes, especially about sunset, is so great as to be almost unbearable; so much so, that many times we were obliged to close our doors and windows. The consequences were, that we suffered severely from malarial fevers, some of them of the most obstinate character. From ten per cent. to forty per cent. of the garrison, were constantly under its influence.

On the opposite side of the bay, about three miles south-east, was Fort Morgan, situated on Mobile Point, a long sandy ridge projecting out across the bay from the east. Malarial influences here were slight, and the garrison at Fort Morgan suffered but little from periodical fevers. The garrison at Fort Morgan consisted of one company, and at Fort Gaines, of two companies, of the 15th U. S. Infantry.

On the 13th of August, an officer of the U. S. Engineer Corps returned to Fort Morgan from New Orleans, where yellow fever was then prevalent, and in a few days was taken sick and died with black vomit. On the 27th, the commanding officer and commissary sergeant, who had attended the engineer officer, were taken, and on the 30th, died. Two other officers were sent down from Mobile, together with the best medical skill, yet within a week, fifteen of the forty odd men, and three of the officers were either dead or had it, every one of whom died except the hospital steward; he was saved by heroic doses of quinia, thirty grains every three or four hours. At this juncture, the remnant of the garrison evacuated the fort, and moved out to "Navy Cove," three or four miles from the fort. No new cases were developed after the removal of the command.

At our fort we had established a strict military quarantine, disinfected all outhouses by means of carbolic acid, whitewashed the buildings, etc., and the result was, that while we suffered so much more severely from malaria than Fort Morgan, yet but one case occurred at Fort Gaines, and that as follows: About the 10th of November, the disease was declared to have disappeared from Mobile, and during the last week in November, a general court martial was ordered to sit in Mobile. Among others (from Fort Gaines), the Post Surgeon and a Sergeant of one of the companies were summoned as witnesses. While this sergeant was in Mobile, he stopped several nights in a house where yellow fever had occurred. On the last day of the month, November, he returned to his post. Next morning, his name appeared on the company's sick list, but as he did not appear personally, he was not excused from duty. Just before sunset of the same day, I was sent for in great haste, and found him in bed, with some delirium, suffused eyes and countenance, severe pain in head and lumbar region, and vomiting a whitish flocculent substance quite freely. He was immediately sent to the hospital, placed in a hot mustard bath, and given an active cathartic. He soon became erratic, wanting any and everything, which was no sooner procured than it would be refused. He would get up by the fire, then lie on a bed for a moment, which not suiting him, would be changed for another. In my perplexity and ignorance, I began giving him quinia in gr. v. doses, but about midnight he began to have black vomit. Convulsions set in, and he sank into a comatose condition, and about 4 A.M., he died. Daylight revealed the fact that his face and neck were as yellow as brass. I *then* knew I had been dealing with a case of yellow fever. He was buried as soon as possible, and under instructions of the commanding officer, I burned everything in the ward with which he had come in contact, saturated the ward with a hot solution of carbolic acid and chloride of lime, and burned camphor on the stove. All this may seem laughable, but should be excused on account of my ignorance as to what should be done, as I had only just begun the study of medicine, and knew only the routine treatment of the more ordinary diseases. The disease did not communicate itself to any other person, which was doubtless owing more to the extremely cold weather prevalent at that time, than to my destructive and disinfecting measure.

My next experience was in fighting yellow fever at a distance; in other words, keeping it from our doors when it approached so near, that with the least cessation of vigilance, it might have obtained a foothold, and decimated our garrison as it had done before, and since. This was at Fort Jefferson, Dry Tortugas. This place is particularly obnoxious to yellow fever, and it has occurred there a number of times. In 1867, it was brought there from Havana, eighty miles south, and carried off forty-two men and several officers before it was arrested. The Post Surgeon, his son, and the Post Commander, had died; a large part of the garrison were down with it when Dr. Mudd, sent there for his complicity in the assassination of President Lincoln, was called upon to do what he could, until other medical assistance could be obtained from Key West, seventy-five miles distant. One of the first acts of Dr. Mudd was, to order one hundred of the ten-inch columbiads—the full charge of powder for which is forty pounds—to be loaded, and about midnight, they were all fired off in rapid succession, filling the fort with its terrible concussion, and dense sulphurous smoke. As soon as other medical assistance arrived, all of the command able to do so, were moved to Loggerhead Key, three miles west, and the disease rapidly subsided.

Dr. Mudd deserved, and received a great deal of crédit for his efforts under the circumstances, and it was an interesting question whether his action in firing off the cannon, had any effect in staying the progress of the disease. Some maintained that it did.

The *germs* of this epidemic, whereby two-thirds of a garrison of four companies had been attacked, with nearly fifty deaths, were proven before a military commission to have been brought in, or on, the person of an officer, who had visited Havana on the 4th of July, and about eight days after his return, was taken with it, but recovered. From this case, it spread with great rapidity, as many as forty cases having occurred during a single night. Here, malarial fevers are rare. I cannot recall a dozen marked cases during the two years and three months I was there, i. e., from January, 1869, to April, 1871.

On the 22d of May, 1870, yellow fever was declared epidemic in Key West, and as all our communication with the outer world was through that place, upon the recommendation of the Post Surgeon, a rigid quarantine was established; and from the 22d of May until the 10th of November, a period of nearly six months, no vessels were allowed to approach within a mile of the fort, under the penalty of being fired into, and no persons were permitted to come to, or go from, the fort.

All our mails, provisions, etc., were put off by the crew of the vessel on "Bird Key," nearly a mile to the leeward of the fort (the north-east trade winds prevail there); and after the vessel had left the mail matter, was fumigated or disinfected over burning sulphur in a close apparatus devised for the purpose, and then brought, in many instances brown and discolored, to the fort for distribution. Should any provisions or clothing have been landed, a squad of prisoners were sent down with a mixture of whitewash and sulphate of iron, with which every box, barrel, and package was thoroughly coated, and allowed to remain in the hot sun for twenty-four hours, before being brought to the fort for issue.

Within, the fort was whitewashed throughout; the utmost cleanliness was enforced; tar was burned in the various bastions; privy vaults and sewers were washed out, disinfected, and nearly all locked up, outhouses being built outside on the wharves over deep water.

While, therefore, the epidemic at Key West carried off over one-third of the garrison and a large part of the population; appeared on board, and carried off two of the crew of the Government schooner plying between Key West and Fort Jefferson; and also attacked fatally, one of the light-keepers at Loggerhead Lighthouse, three miles west of us, who was so incautious as to go to Key West, although stopping there but a few hours; yet, surrounded as we were by the pestilence, and, as before said, in a place especially obnoxious to it, under the efficient means adopted, and which caused a great deal of discomfort and privation, we entirely escaped it.

Let us reverse the picture for a moment. During the summer of 1873, other medical officers were stationed there. *They* did not believe in the contagion of yellow fever. Oh, no! It depends upon local causes, and will appear independently of importation, and we will have none of your quarantine and police regulations, with all their attending discomforts and privations!

What was the result? Yellow fever was again prevalent in Key West. A child of one of the medical officers was allowed to go there, and soon after her return, was taken with yellow fever; and from this *focus*, it spread among the garrison, at that time consisting of only one small company of fifty or sixty men, and one officer. In a short time, the commanding officer, the hospital steward, his wife, and sixteen or eighteen of the men, were *dead*, and the rest were saved by precipitately evacuating the fort.

Last year, 1873, yellow fever appeared epidemically in Memphis, Shreveport, Pensacola, Key West, Dry Tortugas, and to a considerable extent, in Mobile, New Orleans, and other places.

At that time, I was in the U. S. Marine Hospital at Mobile, and the history of its appearance in Mobile, from the report of the board of health, and my own observations, was substantially as follows: A man by the name of Dixon, a resident of northeast Texas, becoming alarmed at the approach of yellow fever towards his section, thought to fly from it, and in so doing, passed through Shreveport, where it was then raging in a malignant form. He passed on down to New Orleans, on to Mobile, and took a steamer for the "Eastern Shore," a noted summer resort, where he proposed stopping. While on the steamer, he was taken sick, brought back to the city, and carried to the city hospital. This was on the 11th of September. On the 13th, he died from *black vomit*. On the 18th, three *sisters of charity*, who had attended him, were attacked, and died. Before another twenty-four hours had elapsed, a vast number of people fled from the city, crowding and pressing into service every means of conveyance; in fact, panic and demoralization ensued. The city authorities, under the advice of medical men and board of health, established a cordon of police around the *infected* hospital, and filling large street sprinklers with a strong solution of carbolic acid and sulphate of iron, had them driven through the streets in that neighborhood twice a day, saturating the streets and gutters so that the atmosphere was loaded with the fumes of the acid. This solution was made of two gallons of the *crude* carbolic acid, and twenty pounds of the sulphate of iron to the barrel of water.

The Marine Hospital was in close proximity to the City Hospital—next square—and under the advice of the surgeon in charge, several pailfuls of the above solution were daily applied around the hospital, in the privies, and gutters, and drains. Within the hospital, half a pound of the crystalized carbolic acid was dissolved in a pailful of boiling water, and with a brush-broom, sprinkled through all the wards and halls daily. The walls were whitewashed with carbolized whitewash.

Under this regime, the disease was confined almost exclusively to that portion of the city. The wind, for ten or twelve days, blew constantly from the northeast, and the disease spread slowly to the southwest; but being persistently followed by the "carbolic acid brigade," surrounding it with all the antiseptic power possessed by carbolic acid and sulphate of iron, it was, as I shall always believe, *smothered* to a great extent, and prevented from becoming epidemic.

No case occurred in our hospital until the surgeon in charge contracted it, as he believes, by standing in front of the City Hospital late one evening, while it was prevalent there. He was taken on the 9th of October, and recovered in a few days. A friend who was stopping with us, having sent his family North, myself and a colored attendant, attended the surgeon during his illness. On the 16th, our friend was taken sick, and on the 19th, *died* from the disease in its most malignant form, as stated by a physician of upwards of forty years' practice, who was called in consultation. Complete suppression of urine took place thirty-six hours previous to death, and there was copious black vomit. Having been doubly exposed in administering to the wants of the two foregoing cases, I was the next seized, and I propose to give the symptoms, treatment, and progress *in extenso*.

About 9 P.M., October 19th (about four hours after the death of our friend), I was seized with rigors; cold flashes played up and down the spinal column, seeming like the slapping of a towel saturated with ice-water, upon it. Apprehending the onset of yellow fever, I took thirty grains of quinia, a hot pediluvium, and went to bed. An intense headache set in, so that I could not rest, and at midnight I got up and sat by the fire until morning, when I proceeded about my duties. So long as I kept *going*, I would partially forget that anything was wrong; stopping for a moment, it would seem as though every organ in the body was at odds and ends. About noon, feeling satisfied yellow fever had me in its grasp, I set about preparing for it. First, thirty grains hydrarg. mass was given me; two hours after, a large saline cathartic, and an enema. As soon as the bowels moved, thirty grains quinia, a hot mustard bath, then placed in bed, rolled up in several heavy blankets, a fire built, bed drawn up to it, and hot bricks rolled up in towels wrung out in hot water, and placed at the small of the back and feet. I then had intense headache, excruciating pain in lumbar region, suffused eyes and countenance, a tongue coated with a whitish fur, apparently an eighth of an inch thick, and quick pulse.

The fæcal discharges at first were natural, but soon became dark; and finally, black and ropy like cold tar, with a very fetid odor, adhering to the chamber like glue. My headache then left me. During the succeeding eighty hours, the treatment pursued was: Quinia, gr. v.; acid sulph. dil. *m.* xx. every two hours. The hot bricks were constantly changed as fast as they became cool; virtually, making a "*sweat-box*" under the seven or eight blankets. With an occasional draught of warm orange-leaf tea, causing copious perspiration, which was encouraged by all possible means. On the second day my stomach became quite irritable, but a dose of the acid quinia mixture seemed grateful to it, and by turning on the right side, I could almost imagine I felt the irritating material pass out through the pyloric orifice. In the course of the attack, I had several evacuations of the black fetid tenacious stools. I did not close my eyes or lose my consciousness until about 3 A.M. of the fourth day, when the disease seemed suddenly to leave me, and I fell into a sound sleep and slept until the afternoon, when I woke up, feeling refreshed and able to get up and go about my business, but kept my bed as a matter of precaution until the 27th, when I got up and went down stairs, and next day resumed my duties. As soon as convalescence was established, a voracious appetite manifested itself, and my tongue, which, in the meantime had become black, begun to exfoliate in patches and shreds, disclosing the raw and "beefy" tongue underneath the coating; and several days after when I saw my wife, she laughed at its ludicrous appearance. It was nearly two weeks after I had gone about my occupation, before it was entirely clear of the coating.

I paid particular attention to the condition of my pulse. During the first twenty-four hours, it was weak, thready, and compressible. During the second and third days, it gradually fell to 55, 50, and lower, and this condition alarmed me. I imagined I might die from sheer stoppage, if it kept on going down. This condition of the pulse could not be attributable to the sedative effect of the quinia (for I believe quinia in large and repeated doses has a sedative influence), because, while ordinarily a moderate use of quinia will, with me, produce fullness of the head, vertigo, tinnitus aurium, or almost total deafness; yet in this instance, although I had taken upward of half an ounce during four days and nights, it did not have any such effects, showing the large quantity taken was neutralized by the poison.

The same day convalescence was established in my case, one of the men who had nursed me was taken, and in all, nine cases occurred in our hospital; but, as heavy frosts had fallen during my attack, or in consequence of the antiseptic means again carried out, they were of a mild form, and all recovered under, essentially, the same treatment.

I took altogether, as before said, rather more than ℥ss. of the quinia, and ℥ij. of the acid sulph. dil., and I think this treatment to be the most correct, although many, if not most authors, decry the use of quinia in large doses. I do so upon the following hypothesis: According to Aitken, "Ammonia is universally present in the black vomit and breath; the urea of the suppressed urine is transformed by metamorphosis into carbonate of ammonia." The acid was given under the idea of neutralizing the tendency of the blood to become alkaline under this condition. The quinia was given with a view to counteract or destroy the poison. Quinia, which comes as near being a specific as anything we have in our *Armamentarum Medicamentum*, I have for a long time believed to act as an antiseptic. Calvert, I believe, has demonstrated, that a solution of quinia possesses the property of destroying certain minute forms of animal life, and I believe no harm can result from the use of quinia, however large or often repeated the dose may be, so long as the poison is not fully neutralized by it.

Again, believing the essential pathological lesion to occur in the kidneys, it was endeavored by the hot mustard baths, blankets, and hot bricks, to induce the skin to act vicariously for the kidneys. That it did, seemed confirmed by the strong ammoniacal odor given off by the skin, during the copious perspiration induced by these means. Diuretics are absolutely useless; their administration will only be adding fuel to the flames in the congested kidneys.

I have in the above narrative stated what I have observed, in part, in regard to yellow fever, from which I have come to the following conclusions: That it is *contagious*. In support of this, I cite the case of Fort Jefferson, in 1867 and 1873; Fort Morgan in 1867; Mobile in 1873. In all these instances, the *importation* on the *person*, and its propagation therefrom, was as clear as any event that can occur among men. In each of these instances, certain parties had been exposed to yellow fever at some distant point; arriving at their posts, or places where it was not, were, after a short period of incubation, seized with it; the next to be attacked would be those attending them, and so on. Let me cite another instance: While the surgeon of the hospital at Mobile was ill, five seamen from the ship "W. A. Campbell," just in from Liverpool, came to the hospital suffering from rheumatism or venereal diseases. Not having their hospital permits properly authenticated, I took them into the surgeon's room, in order that he should decide as to their admission or not. They were probably in the room five minutes. Several days after, *three* of the five were almost simultaneously attacked with yellow fever. I do not understand the term *contagion*, unless these instances establish it in a measure. It is not necessary that a person be brought in immediate contact with those having yellow fever in order to be infected, any more than in case of small-pox, which all admit *is* contagious, yet one of our professors has in his lecture, related an instance where small-pox was communicated from the burning of an infected bed, to an invalid several hundred feet distant. It is certain, however, that the radius of contagion of yellow fever is extremely limited. It does not militate against the contagion of yellow fever to say, that because *some* will be infected, others will not, who have been equally exposed. The same phenomenon is seen in all known contagious diseases — scarlatina, *par excellence*—nor that it occurs sporadically as well as epidemically. This is also true of the admitted contagious diseases. There are other necessary elements requisite for the propagation of yellow fever, as well as other contagious diseases, besides simple contact. In this case, the principal one seems to be an accumulation of animal matter, and the exposure of the same to the surrounding atmosphere. The drainage of our southern cities is notoriously bad; mostly superficial, and is of little consequence in the removal of the accumulations of years. The privies are shallow pits, with no outlet; and if cleaned at all, the contents are necessarily exposed to the atmosphere by the night scavengers. Therefore, I believe, that if the removal of all animal matter, or its thorough disinfection or destruction could be accomplished in our southern cities, yellow fever would never present its former malignancy and fatality. While this cannot or will not be done, I am firmly convinced that the free use of carbolic acid will prevent yellow fever from becoming epidemic. This has been done in New Orleans for several years; where, on the appearance of yellow fever, its *foci* has been instantly surrounded by the "carbolic acid brigade," with the result, at least, if not of hemming it in on all sides, destroying its virulence.

As to the connection of yellow fever with malaria, I have shown, in the cases of Forts Morgan and Jefferson, with but little malarial influences, it has displayed all its destructive effects; while at other places, with overpowering exhalations of miasm, it does not appear. Moreover, I have shown, in all the instances related, that it owed its origin to importation on the person of some one or other. It has been said, that it has been carried in the hold of a ship from an infected port to another, where it has not been previously—that is, a section of infected atmosphere has been locked up, so to speak, in the hold of a vessel, and carried to another port —even across the Atlantic, and upon liberation, propagated the disease. But these ships are navigated by human beings, and unless some of those on board have had it at the port of infection, or during the voyage, I doubt its propagation at any port free from it; consequently, a vessel coming from an infected port, unless she should have had cases on board, is never put in quarantine, and so the theory that it propagated through the person, is an admitted fact.

Finally, should it ever be my fortune to be located in a place liable to the irruption of yellow fever, I would recommend the removal of all animal matter, or its thorough disinfection by means of carbolic acid. In case of its appearance, I would make free use of it in and around all buildings where it may exist, with as near absolute non-intercourse as practicable. I believe carbolic acid holds an almost unlimited power over the germs of yellow fever, and in many respects, is one of the greatest additions to our stock of therapeutical agents in other diseases, and causes of disease, as well as the specific one under consideration.

In the treatment, I would adopt that indicated, to-wit: The free evacuation of the alimentary canal; the free use of quinia; every means in our power of hastening and promoting active diaphoresis, and especially good nursing.

There are many interesting facts in regard to yellow fever, to be gleaned from "the books," by which this paper could have been extended, *ad infinitum;* but as they are accessible to all, I close without reference to them.

